# Activation of CD147 with Cyclophilin A Induces the Expression of IFITM1 through ERK and PI3K in THP-1 Cells

**DOI:** 10.1155/2010/821940

**Published:** 2010-08-16

**Authors:** Ju-Young Kim, Ho Kim, Kyoungho Suk, Won-Ha Lee

**Affiliations:** ^1^School of Life Sciences and Biotechnology, Kyungpook National University, Daegu 702-701, Republic of Korea; ^2^Department of Pharmacology, School of Medicine, Kyungpook National University, Daegu 702-701, Republic of Korea

## Abstract

CD147, as a receptor for Cyclophilins, is a multifunctional transmembrane glycoprotein. In order to identify genes that are induced by activation of CD147, THP-1 cells were stimulated with Cyclophilin A and differentially expressed genes were detected using PCR-based analysis. Interferon-induced transmembrane 1 (IFITM1) was detected to be induced and it was confirmed by RT-PCR and Western blot analysis. CD147-induced expression of IFITM1 was blocked by inhibitors of ERK, PI3K, or NF-*κ*B, but not by inhibitors of p38, JNK, or PKC. IFITM1 appears to mediate inflammatory activation of THP-1 cells since cross-linking of IFITM1 with specific monoclonal antibody against it induced the expression of proinflammatory mediators such as IL-8 and MMP-9. These data indicate that IFITM1 is one of the pro-inflammatory mediators that are induced by signaling initiated by the activation of CD147 in macrophages and activation of ERK, PI3K, and NF-*κ*B is required for the expression of IFITM1.

## 1. Introduction

Interferon-induced transmembrane (IFITM/Mil/fragilis) proteins, originally described based on their expression after IFN treatment [1], belong to a superfamily that is characterized by the presence of two transmembrane domains and an intervening highly conserved intracellular loop. Over 30 members of this superfamily are known to be involved in antiviral defense, immune cell signaling, cell adhesion, oncogenesis, and germ cell maturation [2–6]. As the first identified member of this superfamily, IFITM1 (CD225) has been studied for its involvement in the inhibition of viral replication [7], promotion of cancer cell invasion [8], and expression in transformed cells as a cancer marker [9–12]. 

CD147 (EMMPRIN/basigin/HAb18G/neurothelin/M6/TCSF) has two immunoglobulin-like extracellular domains and a short (39 amino acids long) intracellular domain [13]. CD147 plays a critical role in many pathological and physiological processes in a variety of cell types such as cancer cells, leukocytes, fibroblasts, and endothelial cells [14–17]. Stimulation of CD147 in fibroblast and endothelial cells has been shown to facilitate tumor invasion, metastasis, and angiogenesis [17, 18]. On the other hand, stimulation of CD147 in leukocytes leads to the enhancement of a variety of inflammatory processes that are associated with atherosclerosis, lung injury, rheumatoid arthritis (RA), chronic liver disease, and heart failure [19–21]. Two members of cyclophilin family, cyclophilin A and B (CypA and CypB), can interact and stimulate CD147 [22, 23]. These cyclosporine binding proteins can be secreted in response to inflammatory stimuli. CypA can be secreted from activated platelets, smooth muscle cells (SMCs), and macrophages [24–26]. CypA exerts cytokine-like activities [25, 27] which has been recognized in the pathogenesis of various diseases including rheumatoid arthritis [28–30], sepsis [31, 32], and atherosclerosis [21, 25, 27, 33, 34]. CypB is known to be present in the ER of all cell types and is involved in various functions such as chemotaxis, hepatitis C virus replication, immunosuppression, prolactin signaling, and association with collagen [35–40]. CypB has been found to be present in human blood, milk, and culture supernatant of chondrocytes [41, 42], suggesting that it can also be secreted.

 In order to identify molecules which are induced by the stimulation of CD147, the human macrophage-like cell line, THP-1, was stimulated with CypA. IFITM1 was identified to be one of the molecules that are induced by CypA. Signaling pathways responsible for the expression of IFITM1 and possible role of IFITM1 in macrophage activity were investigated.

## 2. Materials and Methods

### 2.1. Monoclonal Antibodies, Cell Lines, and Reagents

Recombinant human CypA was purchased from BIOMOL International (USA). mAb for CD147 (clone MEM-M6/1) was from Abcam (MA, USA) anti-IFITM1 mAb was purchased from Abnova. PD08059 and U0126 were originated from Cell Signaling (USA); SB203580, Ro-31-8425, JNK inhibitor I (JNK-I1), a cell-permeable fusion protein containing 20 AA of the JNK-binding domain of islet-brain and HIV-TAT_48–57_ [43], and its negative control containing only HIV-TAT were obtained from Calbiochem International Inc. (USA), LY294002 were purchased from Sigma. *α*-actin specific mAb (1A4) was purchased from DAKO (Glostrup, Denmark). Human monocytic leukemia cell line THP-1 [44] was obtained from the American Type Culture Collection (USA). 

### 2.2. Gene Fishing Analysis

Differentially expressed genes (DEGs) were screened by the annealing control primer-(ACP)-based PCR method using the GeneFishing DEG kits (Seegene, Seoul, South Korea) according to a protocol provided from the manufacturer [11]. The amplified PCR products were separated in 2% agarose gel stained with ethidium bromide. The differentially expressed bands were extracted from the gel using the GENCLEAN II Kit (Q-BIO gene, Carlsbad, CA), cloned into a TA cloning vector (Invitrogen, Karlsruhe, Germany) and sequenced. Resulting sequences were compared with GenBank database using the Basic Local Alignment Search Tool (BLAST) search program at the National Center for Biotechnology Information [12].

### 2.3. RT-PCR

Five micrograms of total RNAs isolated from cells were treated with RNase free DNase (BD-Pharmingen), and then used to generate first-strand cDNAs using RevertAid first-strand cDNA synthesis kit with 500 ng oligo (dT)_12–18_ primers. PCR primers were designed with ABI PRISM Primer Express 2.0 (Applied Biosystems) and made by Geno Tech Corp (Korea). For real time RT-PCR reactions, primers were designed for 195 bp, 65 bp, and 51 bp of IFITM1, IFI27, and GAPDH PCR products. Primer sequences were 5′tcatcctgtcactggtattcggctc3′(forward) and 5′gtgggtataaactgctgtatctaggg3′ (reverse) for IFITM1, 5′tctgcagtcactgggagcaa3′ (forward) and 5′cccaatgga gcccaggat3′ (reverse) for IFI27 and 5′tgggctacactgagcaccag3′ (forward) and 5′gggtgtcgctgttgaagtca3′ (reverse) for human GAPDH. Real-time PCR reaction was performed in ABI PRISM 7300 sequence detector (Applied Biosystems) using SYBR green PCR mix (Applied Biosystems) with cDNA corresponding to 125 ng of original total RNA and 400 nM primers in a 20 *μ*L volume. The threshold cycle (Ct) values for IFI27 and IFITM1 reactions were normalized with Ct value from GAPDH reactions. The specificity of the PCR reaction was confirmed by control reactions such as PCR reaction with templates processed without reverse transcriptase and PCR reaction without template. For conventional RT-PCR analysis same process was used for the preparation of cDNA. Primers were designed for 412 bp, 397 bp, and 391 bp of IFITM1, IFI27, and GAPDH PCR products. Primer sequences were 5′ttcactcaacacttccttcc3′ (forward) and 5′actagtaaccccgtttttcc3′ (reverse) for IFITM1, 5′actctggaatgccacggaat3′ (forward) and 5′gagctagtagaacctcgcaacccaa3′ (reverse) for IFI27 and 5′atcactgccacccagaagac3′ (forward) and 5′tgagcttgacaaagtggtcg3′ (reverse) for human GAPDH. After the PCR reaction, the PCR products were run on 2% agarose gel to confirm the size and purity of the PCR products.

### 2.4. Cell Stimulation, Western Blot Analysis, and Gelatin Zymogram

THP-1 cells were stimulated by adding 1 *μ*M of recombinant human CypA or adding 1–30 *μ*g/mL anti-CD147 or anti-IFITM1 mAb in a soluble form. Cell lysates were prepared at appropriate times after activation in 100 *μ*L of triple-detergent lysis buffer. Western blot analysis was performed as described previously in [45]. For the detection of MMP-9 using gelatin zymogram, culture supernatants were collected 24 hours after activation. The MMP-9 activity in the culture supernatant was determined by substrate gel electrophoresis as described previously in [46].

### 2.5. Statistical Analysis

Statistical significance of differences was evaluated by means of a two-sided Student's *t*-test, assuming equal variances. Differences were considered significant when *P* < .05.

## 3. Results and Discussion

### 3.1. GeneFishing Analysis Identified IFITM1 as One of the Proteins that Are Induced after CypA Treatment in THP-1 Cells

**Figure 1:**
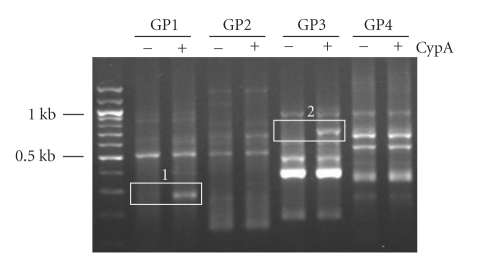
GeneFishing analysis after CypA treatment in THP-1 cells revealed multiple differentially expressed genes. THP-1 cells were treated with or without 0.1 *μ*M of CypA. Total cellular RNAs were isolated 20 hr after stimulation and differentially expressed gene (DEG) levels were analyzed by PCR using 20 different ACS random primer sets (GP1 through GP20). Results for GP1 through GP4 are shown in the figure. White boxes indicate the bands that were analyzed for identification through cloning and sequencing.

**Figure 2:**
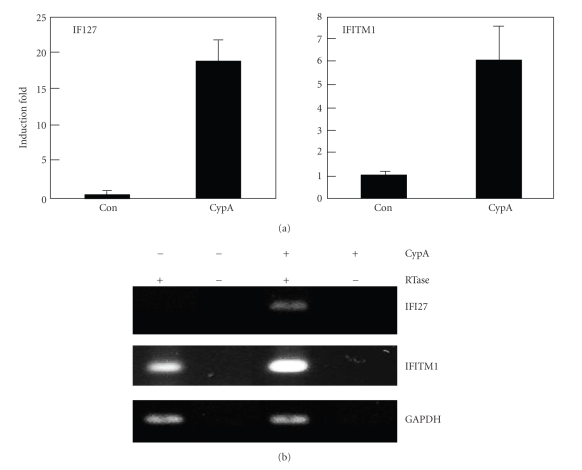
Stimulation of THP-1 cells with CypA induced the expression of IFI27 and IFITM1. (a) THP-1 cells were stimulated with 0.1 *μ*M of CypA for 20 hr. Total cellular RNA was then isolated for real-time RT-PCR with primers specific for IFI27, IFITM1, or GAPDH. Ct values obtained from IFI27 or IFITM1 amplification curves were normalized with that of GAPDH. (b) THP-1 cells were stimulated and cellular RNAs were isolated as in (a). RT-PCR analysis was performed with primers specific for IFI27, IFITM1, or GAPDH and the products were run on agarose gel. These experiments were repeated three times with essentially the same results.

Treatment with CypA has been shown to be able to stimulate THP-1 cells resulting in the production of proinflammatory mediators such as MMP-9, IL-8, TNF-*α*, MCP-1, and IL-1*β* [28]. In order to identify genes that are expressed by CypA treatment, THP-1 cells were stimulated with CypA for 24 hours and the genes showing differential expression patterns were detected using GeneFishing differentially expressed gene (DEG) system. Total RNA extracted from THP-1 cells stimulated with or without CypA were used for the synthesis of cDNA. DEGs were screened by an annealing control primer-based PCR method [47]. Twenty different primer sets were tested which revealed multiple bands with differential expression patterns. Two of these bands (Figure 1, number 1 and 2) were extracted and sequenced for the identification of the corresponding genes. Band number 1 was identified to be homosapiens interferon, alpha-inducible protein 27 (IFI27) (gene bank accession number BC015492) and band number 2 was identified to be human interferon-inducible protein 9–27 (IFITM1) (gene bank accession number J04164). The expression of both IFI27 and IFITM1 is previously known to be induced by interferon. In order to confirm the expression of these genes, RT-PCR analysis was performed after stimulation of THP-1 cells with CypA (Figure 2). Both real-time and conventional RT-PCR demonstrated the induction of both IFI27 and IFITM1 after CypA treatment. In case of IFI27, basal expression levels were not detectable while the low basal expression of IFITM1 was detected.

### 3.2. Stimulation of CD147 with Monoclonal Antibodies Also Activated the Expression of IFITM1 in THP-1 Cells

**Figure 3:**
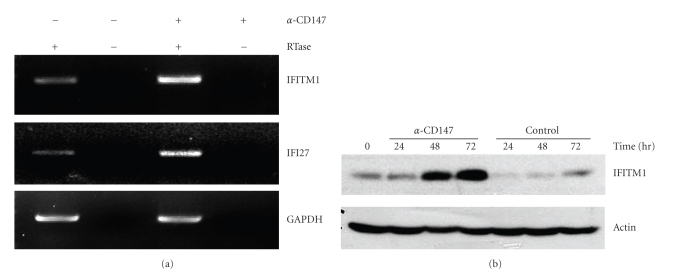
Stimulation of THP-1 cells with anti-CD147 mAb induced the expression of IFITM1. (a) THP-1 cells were stimulated with 10 *μ*g/mL of anti-CD147 mAb for 24 hr. Total cellular RNA was isolated, and RT-PCR analysis was performed with primers specific for IFITM1, IFI27, and GAPDH. (b) THP-1 cells were stimulated with 10 *μ*g/ml of anti-CD147 mAb or isotype matching mouse IgG (control). Cell lysates were obtained at indicated times for the Western blot analysis using mAbs specific for IFITM1 and actin. These experiments were repeated more than three times with essentially the same results.

CypA is known to exert its activity through its interaction with CD147. CD147 expression has been detected in various cell types including cancer cells, leukocytes, fibroblasts, and endothelial cells, and the stimulation of macrophage CD147 with either CypA or anti-CD147 mAb generated cell signaling which was mediated by PI3K and/or ERK [14–17, 29, 33, 48, 49]. Activation of these signaling mediators eventually leads to the activation of NF-*κ*B, the major proinflammatory transcription factor. Activation of macrophage CD147 resulted in the induction of the expression of various genes including cytokine genes and matrix degrading enzymes. In order to demonstrate that the stimulation of CD147 induces the expression of IFITM1, THP-1 cells were stimulated with anti-CD147 mAb. IFITM1 and IFI27 expression was detected to be induced by anti-CD147 treatment through RT-PCR (Figure 3(a)) as well as Western blot analysis (Figure 3(b)). Protein levels of IFITM1 started to be upregulated from 48 hours after activation and continued to increase up to 72 hrs. 

 The clone of anti-CD147 mAb used in the current study is somewhat unique in that it is able to stimulate CD147 to induce signaling. The majority of anti-CD147 mAb clones, however, tend to block the activation. It is possible that the clone of antibody used in the current study may interact with an epitope that can induce conformational change in the CD147 in such a way that it can activate downstream signaling adaptors. It is also possible that cross-linkage of CD147 that is induced by the current antibody may result in an arrangement of downstream adaptor molecules in such a way that facilitates generation of activation signals.

### 3.3. The CD147-Mediated Expression of IFITM1 Required ERK and PI3K as Signaling Adaptors and NF-*κ*B as a Transcription Factor

**Figure 4:**
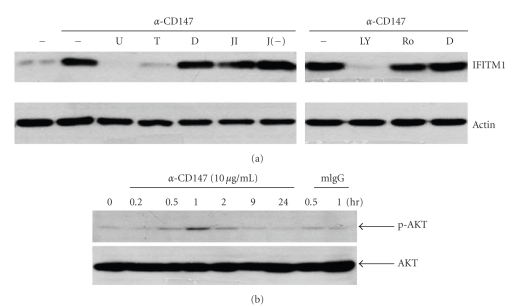
Inhibitors of ERK, NF-*κ*B, and PI3K blocked the CD147-mediated activation of IFITM1 expression. (a) THP-1 cells were preincubated with 10 *μ*M of U0126 (U), 5 *μ*M of TPCK (T), 0.2% of DMSO (D), 10 *μ*M of JNK inhibitor (JI), negative control of JNK inhibitor (J(−)) for 30 min, and 20 *μ*M of LY294002 (LY) or 1 *μ*M of Ro-31-8425 (Ro) for 60 min and then stimulated with 10 *μ*g/mL of anti-CD147 mAb for 48 hr. Cell lysates were collected and subjected to Western blot analysis using mAbs specific to either IFITM1 or actin. (b) THP-1 cells were stimulated with 10 *μ*g/mL of anti-CD147 mAb or mouse IgG (mIgG) for indicated times. Cell lysates were collected and subjected to Western blot analysis using mAbs specific to either AKT or phosphor-AKT. These experiments were repeated twice with essentially the same results.

In order to find out the signaling mediators which are responsible for the CD147-mediated expression of IFITM1, THP-1 cells were stimulated with anti-CD147 mAb in the presence of the inhibitors for mitogen-activated protein kinase (MAPK), PKC, PI3K, and NF-*κ*B. There are three members of MAPK family: ERK, p38, and JNK. As shown in Figure 4(a), an inhibitor for ERK (U0126) blocked the CD147-mediated activation of IFITM1 while JNK inhibitor did not. Lim et al. reported that CD147-mediated induction of MMP-1 in fibroblasts is mediated by p38 [50]. However, the activation p38 MAPK was not required in CD147-mediated activation of IFITM1 because p38 inhibitor failed to block the response (data not shown). Inhibitors of PI3K (LY294002) and NF-*κ*B (TPCK) also blocked the CD147-mediated activation of IFITM1 while PKC inhibitor (Ro-31-8425) did not (Figure 4(a)). The involvement of both ERK and NF-*κ*B has already been demonstrated in the induction of MMP-9 expression in THP-1 cells after stimulation of CD147 with either CypA or anti-CD147 mAb [29, 33]. Since activation of PI3K has not been reported previously, it was confirmed by analyzing the phosphorylation of AKT (the main substrate of PI3K) using Western blot analysis. As shown in Figure 4(b), stimulation of CD147 induced phosphorylation of AKT within 30 minutes after activation and the phosphorylation level peaked at 60 minutes after activation. The phosphorylation of AKT was not detected in control samples which are treated with mouse IgG for 30 and 60 minutes. These data indicates that CD147-induced expression of IFITM1 requires activation of ERK, PI3K, and NF-*κ*B. 

Both ERK and PI3K have been recognized as the upstream signaling molecules involved in NF-*κ*B activation in macrophages. ERK is a well-known mediator of inflammation and the ERK-mediated activation of NF-*κ*B has been implicated in the induction of both MMP-9 and IL-8 in THP-1 cells that had been stimulated with agonistic antibodies against the membrane bound form of GITRL, a member of the tumor necrosis factor superfamily (TNFSF) [51]. Furthermore, similar signaling pathway has been observed in bovine glycomacropeptide-induced expression of IL-8, TNF, and IL-1*β* [52] and Cyclophilin A-induced expression of MMP-9 [29]. On the other hand, there are cases where ERK and PI3K separately activate NF-*κ*B. These include the serum amyloid A-induced activation of NF-*κ*B in peripheral blood mononuclear cells (PBMCs) and THP-1 cells [53] and angiocidin-mediated activation of NF-*κ*B in THP-1 cells [54]. Based on these observations in combination with our data, it is highly likely that the CD147-mediated activation of NF-*κ*B occurs via two separate pathways, one involving ERK and the other involving PI3K.

### 3.4. The Stimulation of IFITM1 on the Surface of THP-1 Cells Also Induced Proinflammatory Responses

**Figure 5:**
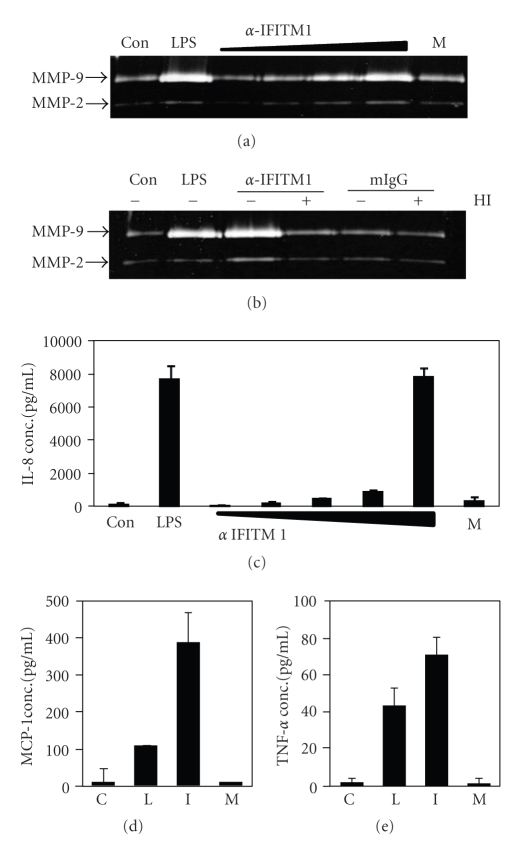
Crosslinking of IFITM1 induces the expression of MMP-9 and IL-8 in THP-1 cells. (a) cells were stimulated with 1 *μ*g/mL of LPS, 0.1, 1, 5, or 10 *μ*g/mL of anti-IFITM1 or 10 *μ*g/mL of isotype matching mouse IgG. Culture supernatants were collected in 24 hr for the measurement of MMP-9 activity using gelatin zymogram. (b) cells were stimulated with 1 *μ*g/mL of LPS, 10 *μ*g/mL of anti-IFITM1 or isotype matching mouse IgG. HI: heat inactivation, 95°C for 2 hr. (c) cells were stimulated with 1 *μ*g/mL of LPS, 0.1, 1, 5, 10, or 30 *μ*g/mL of anti-IFITM1 or 30 *μ*g/mL of isotype matching mouse IgG. Culture supernatants were collected in 24 hr for the measurement of IL-8 concentration using ELISA. (d) and (e) cells were stimulated with 1 *μ*g/mL of LPS (L), 30 *μ*g/mL of anti-IFITM1 (I) or 30 *μ*g/mL of isotype matching mouse IgG (M). Culture supernatants were collected in 24 hr for the measurement of MCP-1 (d) and TNF-*α* (e) concentrations using ELISA. C: control. These experiments were repeated more than three times with essentially the same results.

Involvement of similar signaling molecules for the expression of MMP-9 and IFITM1 raise the possibility that IFITM1 may also have proinflammatory function. In order to test that possibility, IFITM1 expressed on THP-1 cells were cross-linked with specific mAb. As shown in Figure 5(a), treatment with anti-IFITM1 mAb induced the expression of MMP-9 in a dose-dependent manner while same amount of isotype matching mouse IgG did not. The expression of MMP-9 and cytokines from macrophages may have caused by endotoxin which easily contaminate antibody preparations. In order to exclude the possibility of endotoxin contamination, the anti-IFITM1 mAb preparation was treated with heat to inactivate the antibody structure. As shown in Figure 5(b), heat inactivation abolished the stimulatory activity of anti-IFITM1 mAb, thus excluding the possibility of contamination with endotoxin which is heat resistant. Furthermore, stimulation of THP-1 cells with IFITM1 induced expression of IL-8 in a dose dependent manner and treatment with 30 *μ*g/mL of the antibody induced the highest response (Figure 5(c)). At the same concentration of antibody treatment, the expression of other cytokines such as MCP1 and TNF-*α* was also induced (Figures 5(d) and 5(e)). These data indicate that IFITM1 induces proinflammatory responses upon stimulation and cytokines and matrix degrading enzymes are the mediators that can be induced by the activation of IFITM1. 

**Figure 6:**
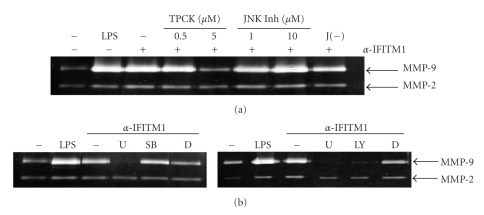
IFITM1-mediated induction of MMP-9 expression requires ERK, PI3K, and NF-*κ*B in THP-1 cells. (a) cells were preincubated with indicated concentrations of TPCK or JNK inhibitor or 10 *μ*M of negative control for JNK inhibitor (J(−)) for 30 min. Cells were then stimulated with 1 *μ*g/mL of LPS or 10 *μ*g/mL of anti-IFITM1 mAb for 24 hrs, and culture supernatants were collected for the measurement of MMP-9 activity using gelatin zymogram. (b) cells were preincubated with 10 *μ*M of U0126 (U), SB203580 (SB), or LY294002 (LY) for 30 min. DMSO (D, 0.1%) was used as a vehicle control. Cells were then stimulated with 10 *μ*g/mL of anti-IFITM1 mAb for 24 hr and MMP-9 activity was tested as in (a). These experiments were repeated twice with essentially the same results.

In order to investigate the signaling pathway induced by IFITM1, THP-1 cells were stimulated with anti-IFITM1 mAb in the presence of various inhibitors of signaling adaptors. As shown in Figure 6, U0126 (ERK inhibitor) blocked the expression of MMP-9 while SB203580 (p38 inhibitor) or JNK inhibitor failed. Treatment with JNK inhibitor, but not with its negative control, tended to enhance the response. This indicates that there could be an interplay between JNK and ERK in IFITM1-mediated cell signaling. Additionally, LY294002 (PI3K inhibitor) blocked the expression of MMP-9. NF-*κ*B is the major transcription factor involved in the expression of MMP-9 during inflammatory activation of macrophages. When TPCK (NF-*κ*B inhibitor) was treated at the same condition, the induction of MMP-9 expression was blocked. These data indicates ERK and PI3K are the downstream mediators of IFITM1-induced signaling in THP-1 cells and activation of these signaling adaptors then leads to the activation of NF-*κ*B for the transcriptional activation of the MMP-9 genes. The involvement of ERK or PI3K in the activation of NF-*κ*B has been documented previously. ERK is a well-known mediator of inflammation and has been demonstrated to be activated in THP-1 cells after inflammatory activation [29, 51, 52]. On the other hand, involvement of both ERK and PI3K in the activation of NF-*κ*B has been shown after stimulation of THP-1 cells with serum amyloid A [53] or angiocidin [54].

In hepatocytes, IFITM1 has been reported to be associated with caveolin-1 and this association enhanced the inhibitory action of caveoin-1 on ERK activation [55]. This discrepancy in the action of IFITM1 with regard to the ERK activity may have been caused by the difference in cell types. It is possible that different adaptor molecules are associated with the intracellular region of IFITM1 in different cell types. It is also possible that the association between IFITM1 and its adaptors is effected by the activation status of the cells. 

Previous data indicated that the stimulation of CD147 also induces the expression of MMP-9 in THP-1 cells through ERK [33]. It is interesting that the CD147-mediated induction of MMP-9 expression and IFITM1-mediated MMP-9 expression use same signaling adaptors in THP-1 cells. In order to test whether stimulation of CD147 and IFITM1 at the same time induces a synergistic expression of MMP-9, THP-1 cells were cotreated with mAbs against these molecules. Simultaneous treatment with these agents, however, failed to induces any synergistic expression of MMP-9 (data not shown). It appears that the addition of one of these agents already induce maximal levels of MMP-9 and cotreatment may not induced further activation since these agents use the same signaling pathway for the induction of MMP-9. 

 Our data provide the first evidence which demonstrates the proinflammatory activities of IFITM1. Previously, IFITM1 has been reported to be enhancing the invasive activity of cancer cells, but the underlying mechanism has not been elucidated [8]. It is possible that the IFITM1-mediated induction of MMP-9 expression and subsequent degradation of extracellular matrix proteins are the molecular mechanism responsible for the enhancement of cancer cell invasion.

## 4. Conclusion

Although CD147 has been implicated in various biological processes associated with normal and pathologic conditions, the exact role of it is not well known at the molecular level, except in a few cases. In myeloid cells including macrophages, CD147 is expressed at high levels and stimulation of it induces strong inflammatory responses such as production of proinflammatory mediators, especially matrix degrading enzymes [28, 33, 56]. In THP-1 cells, high basal level expression of CD147 was detected and treatment with either CypA or anti-CD147 mAb induced expression of inflammatory mediators such as cytokines and matrix degrading enzymes [21, 28, 56]. These activities of CD147 are expected to contribute to the pathogenesis of various diseases where macrophage inflammatory responses play essential roles. In chronic inflammatory diseases such as atherosclerosis, the generation of foamy macrophages is responsible for the formation of fatty streak, the hallmark of the disease [57]. Later, these cells are the main cell type responsible for the formation of atherosclerotic plaques and plaque rupture which leads to the occurrence of acute myocardial infarction [58]. Immunohistochemical analysis revealed the high level expression of CD147 in macrophages in atherosclerotic plaques [33, 34]. CD147 is expected to contribute to the inflammatory activation of macrophages during the pathogenesis of atherosclerosis. Our data provides evidences that IFITM1 is one of the molecules that are induced after CD147 activation. Further analysis demonstrated that IFITM1 itself may perform proinflammatory activities by activating cytokines and matrix degrading enzymes. These data indicate that there are cascade of inflammatory signal where one step of inflammatory activation involving CD147 is amplified into next round of proinflammatory cycle where much high number of proinflammatory mediators, which includes IFITM1, are activated. These vicious cycles of inflammatory cascade are believed to be effective in other diseases where macrophage inflammatory activation is involved. Clearly, development of the therapeutic target for the treatment of these chronic inflammatory diseases requires a concoction of ways to sever this vicious cycle at the early stages of its amplification.
